# Supplementary material for: Mycophagous rove beetles highlight diverse mushrooms in the Cretaceous
Source: Nat Commun. 2017 Mar 16;8:14894. doi: 10.1038/ncomms14894 (PMC5357310; doi:10.1038/ncomms14894)
Supplement: Supplementary Information — Supplementary Figures, Supplementary Tables, Supplementary Notes and Supplementary References. [file ncomms14894-s1.pdf]

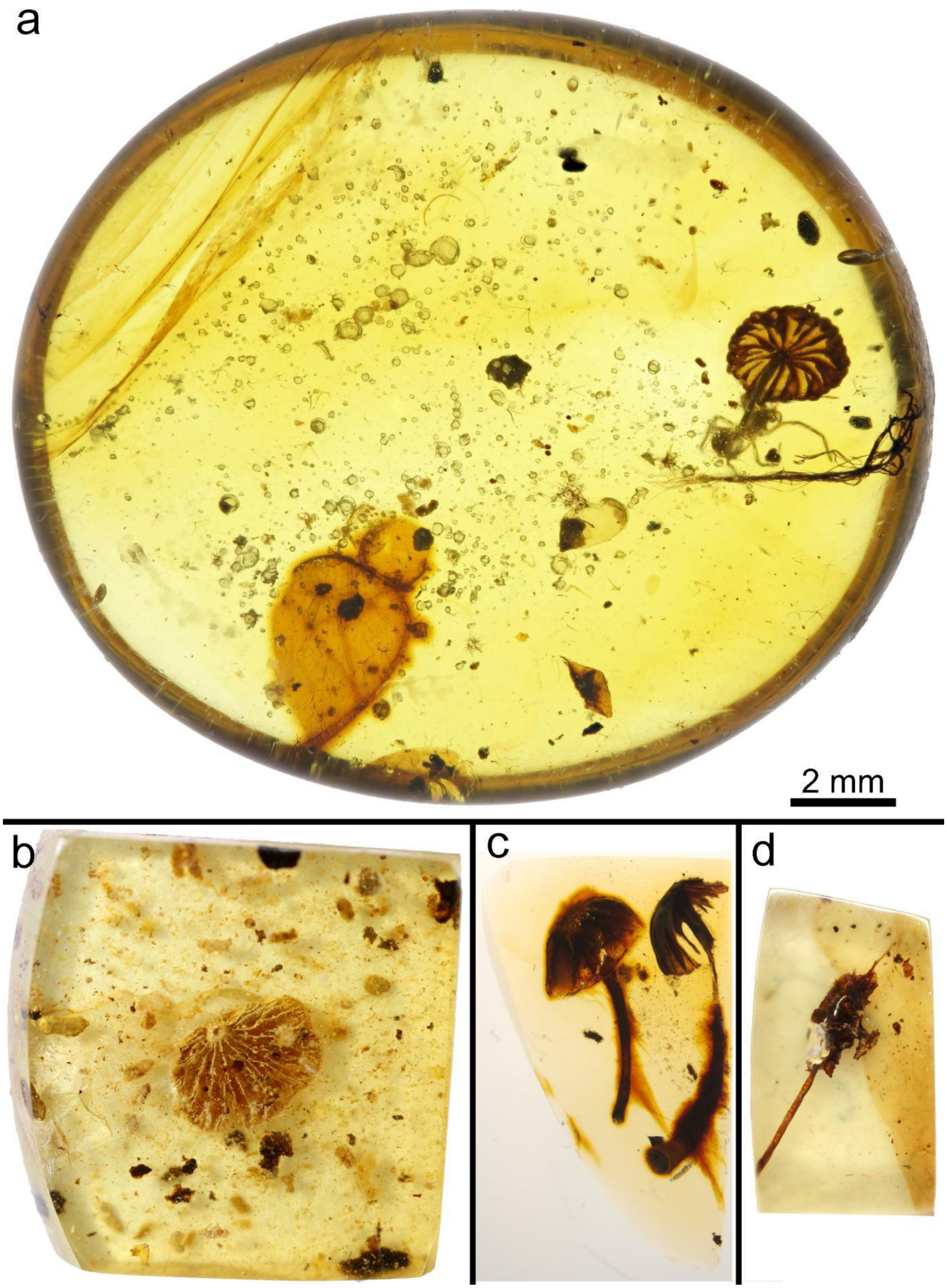

Supplementary Figure 1 | General appearances of mushroom-containing ambers from northern Myanmar. (**a**) Rounded amber piece containing Taxon A, NIGP164520. (**b**) Square-shaped amber piece containing Taxon C, NIGP164523. (**c**) Subtriangular amber piece containing two individuals of Taxon B, NIGP164521 and NIGP164522. (**d**) Rectangle amber piece containing Taxon D, NIGP164524. Scale bar, 2mm.

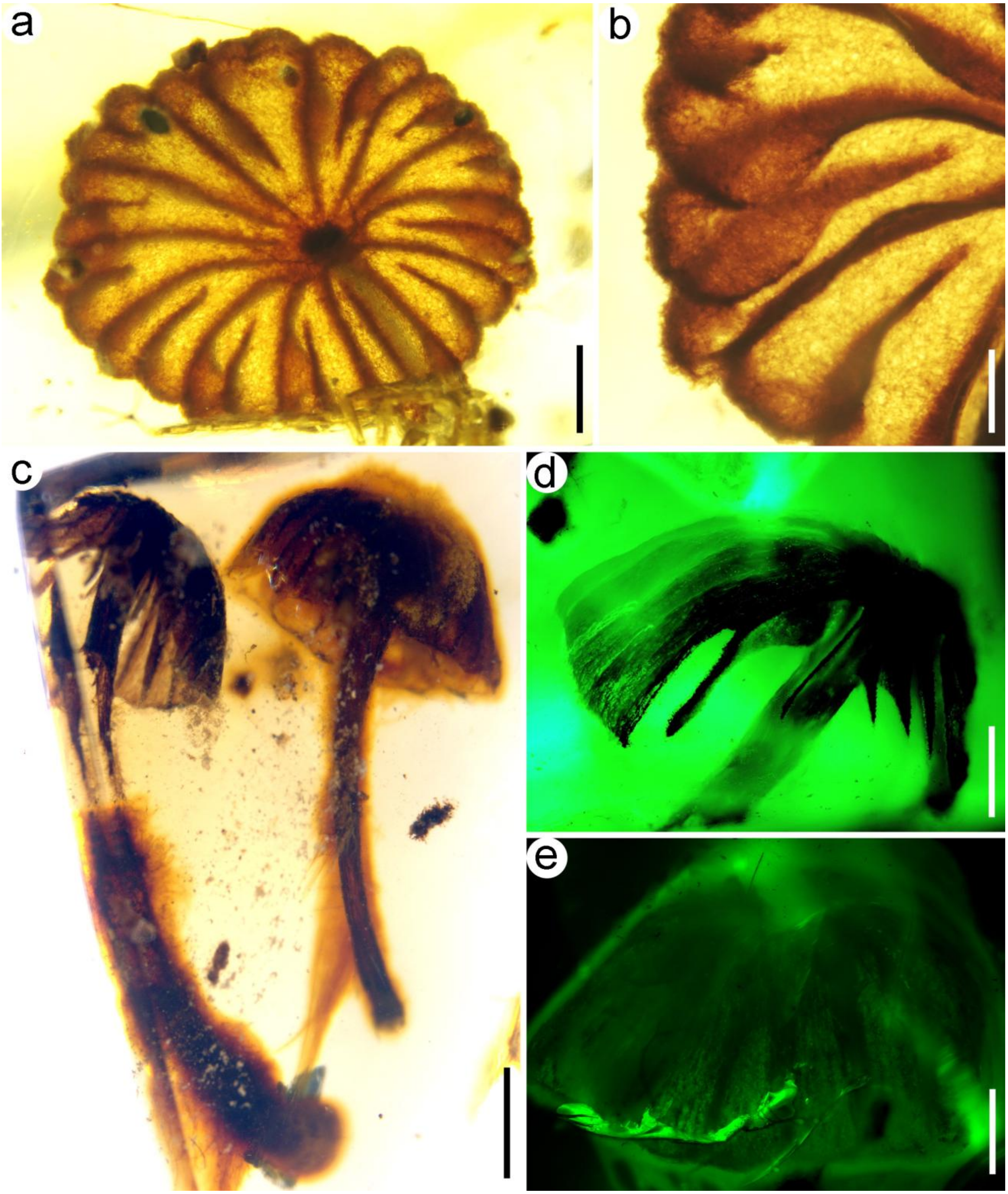

Supplementary Figure 2 | Details of mushrooms in mid-Cretaceous amber from northern Myanmar. **a–c** under normal reflected light; **d** and **e** under fluorescence. (**a**) Top view of Taxon A, NIGP164520. (**b**) Ventral view of Taxon A, showing details of lamellae. (**c**) General appearance of two individuals of Taxon B (NIGP164521 and NIGP164522), showing the strongly plano-convex cap and long stalk. (**d**) Laterally top view of one individual of Taxon B (NIGP164522), showing radially sulcate cap; (**e**) lateral view of the other individual of Taxon B (NIGP164521), showing the median depression on cap. Scale bars, 500 μm in **a**, **d** and **e**; 200 μm in **b**; 1 mm in **c**.

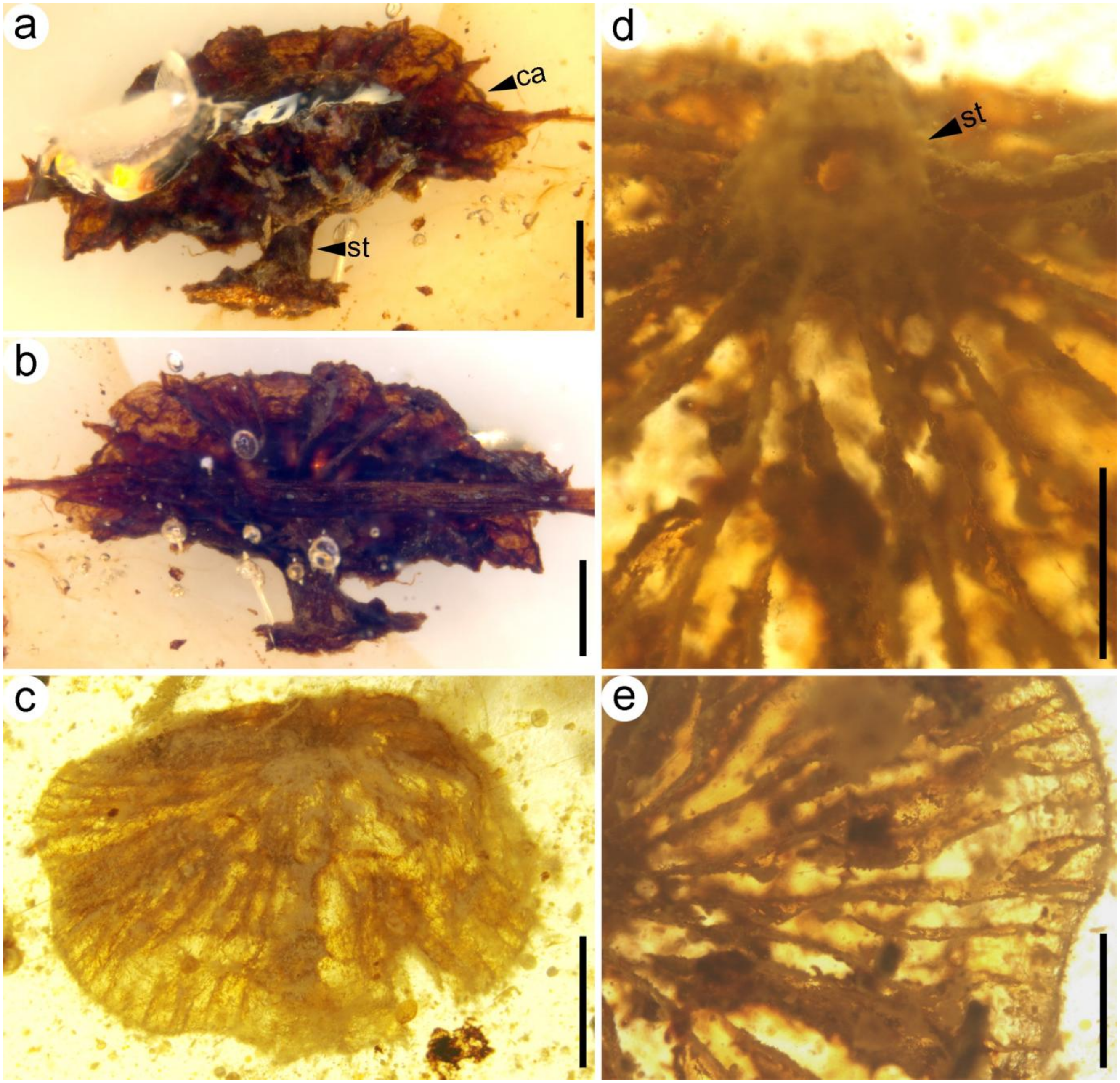

Supplementary Figure 3 | Details of mushrooms in mid-Cretaceous amber from northern Myanmar. (**a**) Top view of Taxon D, NIGP164524. (**b**) Ventral view of Taxon D, NIGP164524. (**c**) Top view of Taxon C, NIGP164523. (**d**) Details of base of stalk and lamellae of Taxon C, ventral view; (**e**) details of lamellae of Taxon C, ventral view. Abbreviations: ca, cap; st, stalk. Scale bars, 500 μm in **a**, **b**, **d** and **e**; 1 mm in **c**.

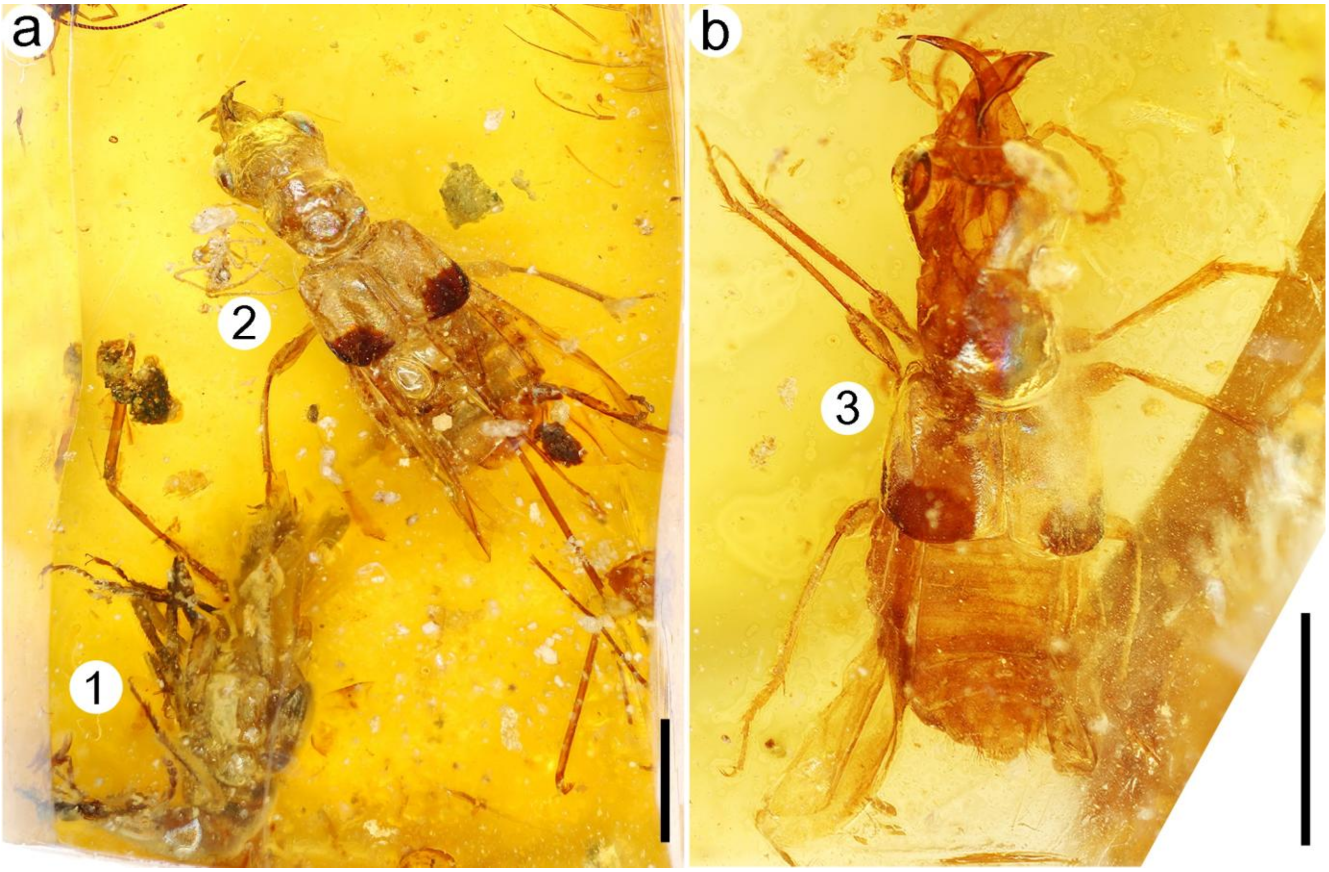

Supplementary Figure 4 | Mycophagous oxyporine rove beetles in mid-Cretaceous amber from northern Myanmar, Taxon 1, preserved in one amber piece prior to preparation. (**a**) Individual 1 (NIGP164525) and individual 2 (NIGP164526) of Taxon 1 preserved together. (**b**) Relatively smaller individual 3 (NIGP164527) of Taxon 1. Scale bars, 2 mm.

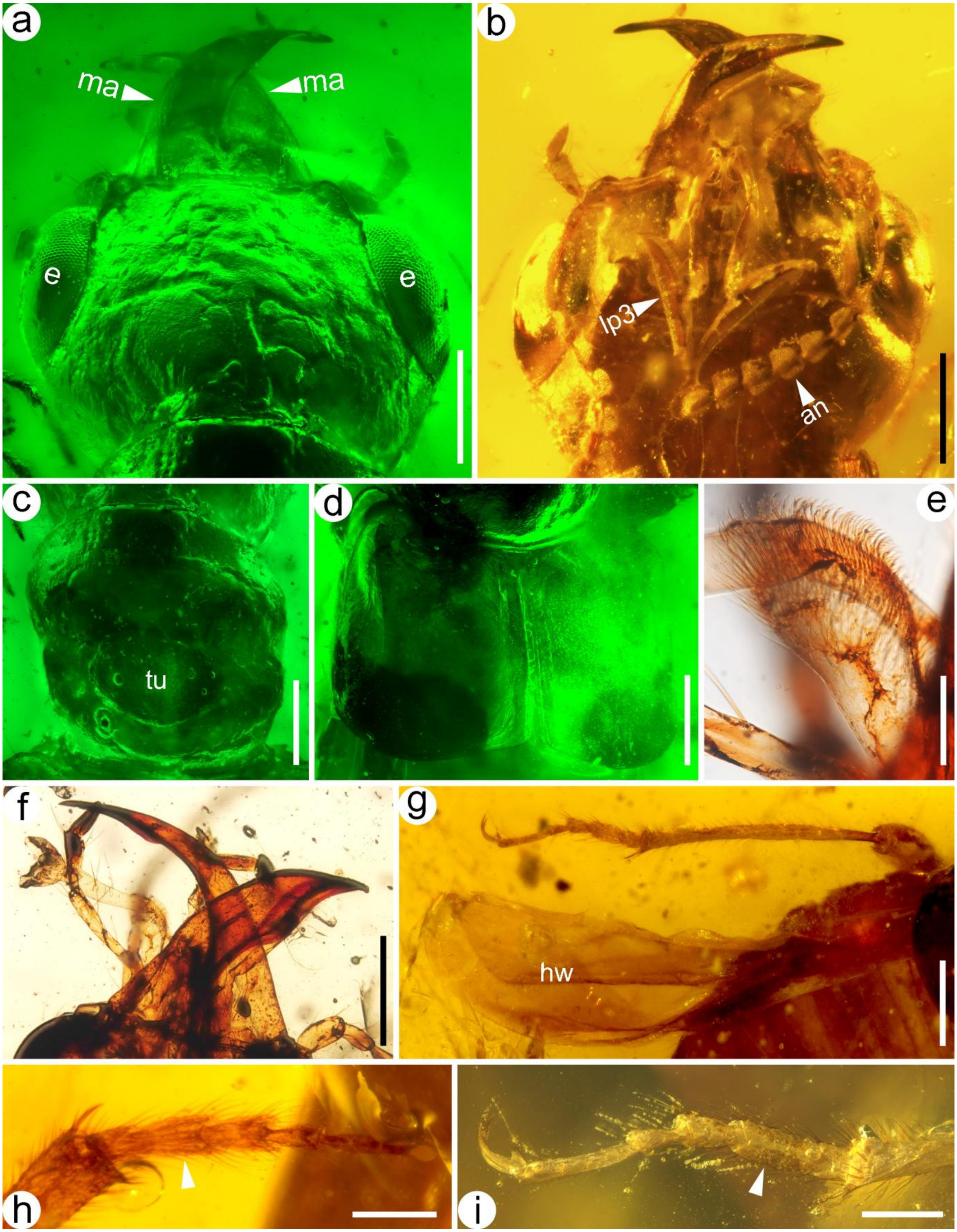
a
ma
ma
e
e
b
lp3
an
c
tu
d
e
f
g
hw
h
i

Supplementary Figure 5 | Details of mycophagous oxyporine rove beetles in mid-Cretaceous Burmese amber, Taxon 1. a, c and **d** under fluorescence, others under normal light. (**a**) Dorsal view of head (individual 2, NIGP164526), showing anteriorly protruding mandibles. (**b**) Ventral view of head (individual 2, NIGP164526), showing specialized labial palpi and characteristic antennae. (**c**) Enlargement of pronotum (individual 2, NIGP164526), showing median tubercle. (**d**) Enlargement of elytra (individual 3, NIGP164527). (**e**) Brush-like pseudomola of mouthparts (individual 3, NIGP164527). (**f**) Mouthparts of individual 3, showing mandibles, maxillary and labial palpi. (**g**) Left hind legs and hing wing of individual 3. (**h**) Right protarsus (individual 3, NIGP164527), with elongate tarsomere 2 indicated. **(i**) Left metatarsus (individual 3, NIGP164527), with elongate tarsomere 2 indicated. Abbreviations: an, antenna; e, eye; hw, hind wing; lp, labial palpomere; ma, mandible; tu, tubercle. Scale bars, 500 μm in **a**–**d**, **f** and **g**; 100 μm in **e**; 200 μm in **h** and **i**.

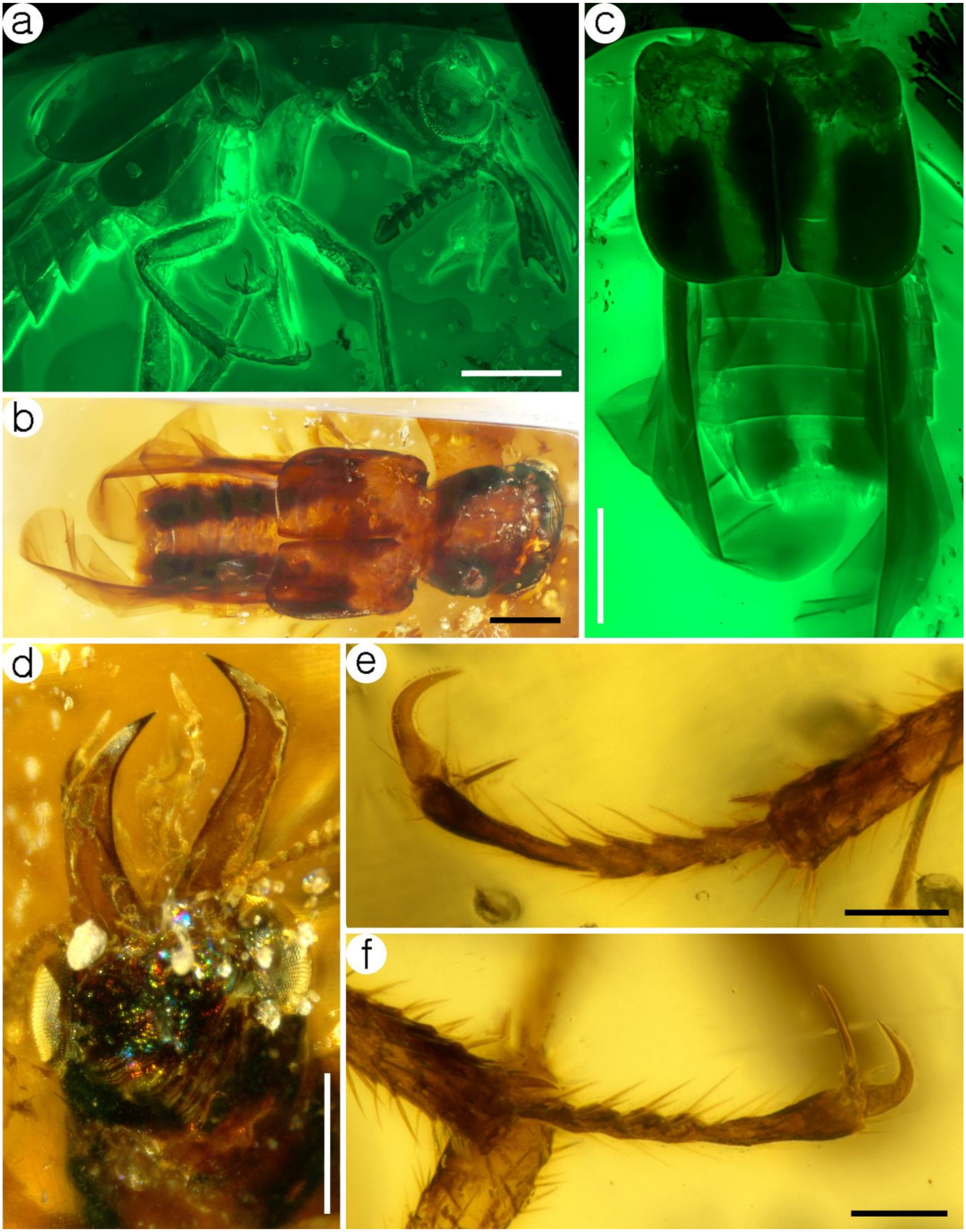
a
b
c
d
e
f

Supplementary Figure 6 | Mycophagous oxyporine rove beetles in mid-Cretaceous amber from northern Myanmar, Taxon 2, NIGP164528. a and **c** under fluorescence, others under normal reflected light. **(a**) Lateral view of head, pronotum, elytra and basal portion of abdomen. (**b**) Dorsal view of pronotum, elytra and abdomen. (**c**) Enlargement of elytra and abdomen, showing colour patterns on them. (**d**) Enlargement of head, showing enlarged and elongate mandibles. (**e**) Left and (**f**) right mesotarsus. Scale bars, 500 μm in **a**–**d**; 100 μm in **e** and **f**.

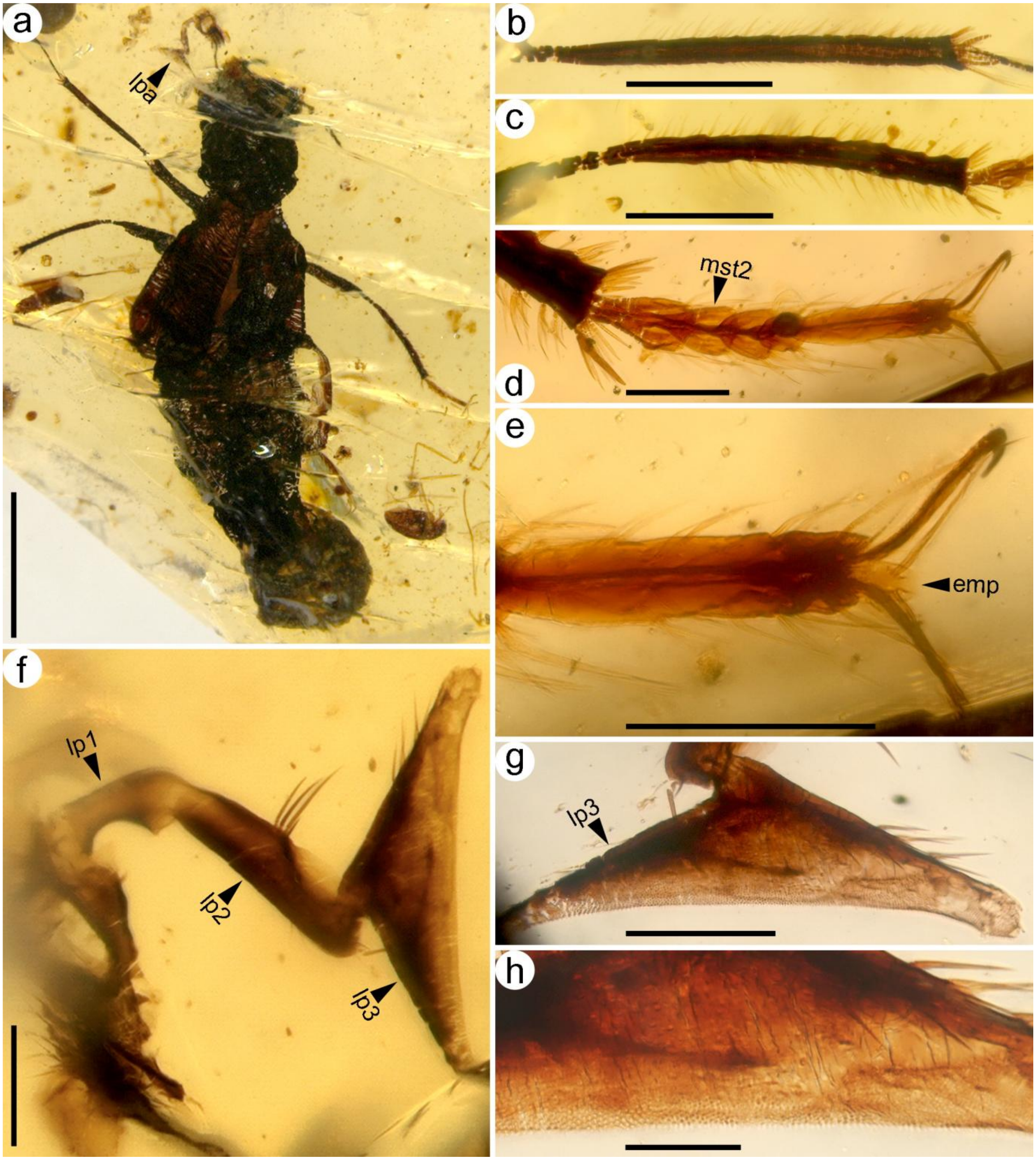

Supplementary Figure 7 | Mycophagous oxyporine rove beetles in mid-Cretaceous amber from northern Myanmar, Taxon 3, NIGP160556, under normal reflected light. (**a**) Dorsal habitus. (**b**) Left protibia. (**c**) Right mesotibia. (**d**) Right mesotarsus. (**e**) Enlargement of **d**, showing apical tarsomere, elongate claw and empodium. (**f**) Left labial palpus. (**g**) Enlargement of **f**, showing apical palpomere. (**h**) Enlargement of **g**, showing dense fine dot-like sensory organs covering its apex. Abbreviations: emp, empodium; lp, labial palpomere; lpa, labial palpus; mst, mesotarsomere. Scale bars, 2 mm in **a**; 500 μm in **b** and **c**; 100 μm in **h**; 200 μm in others.

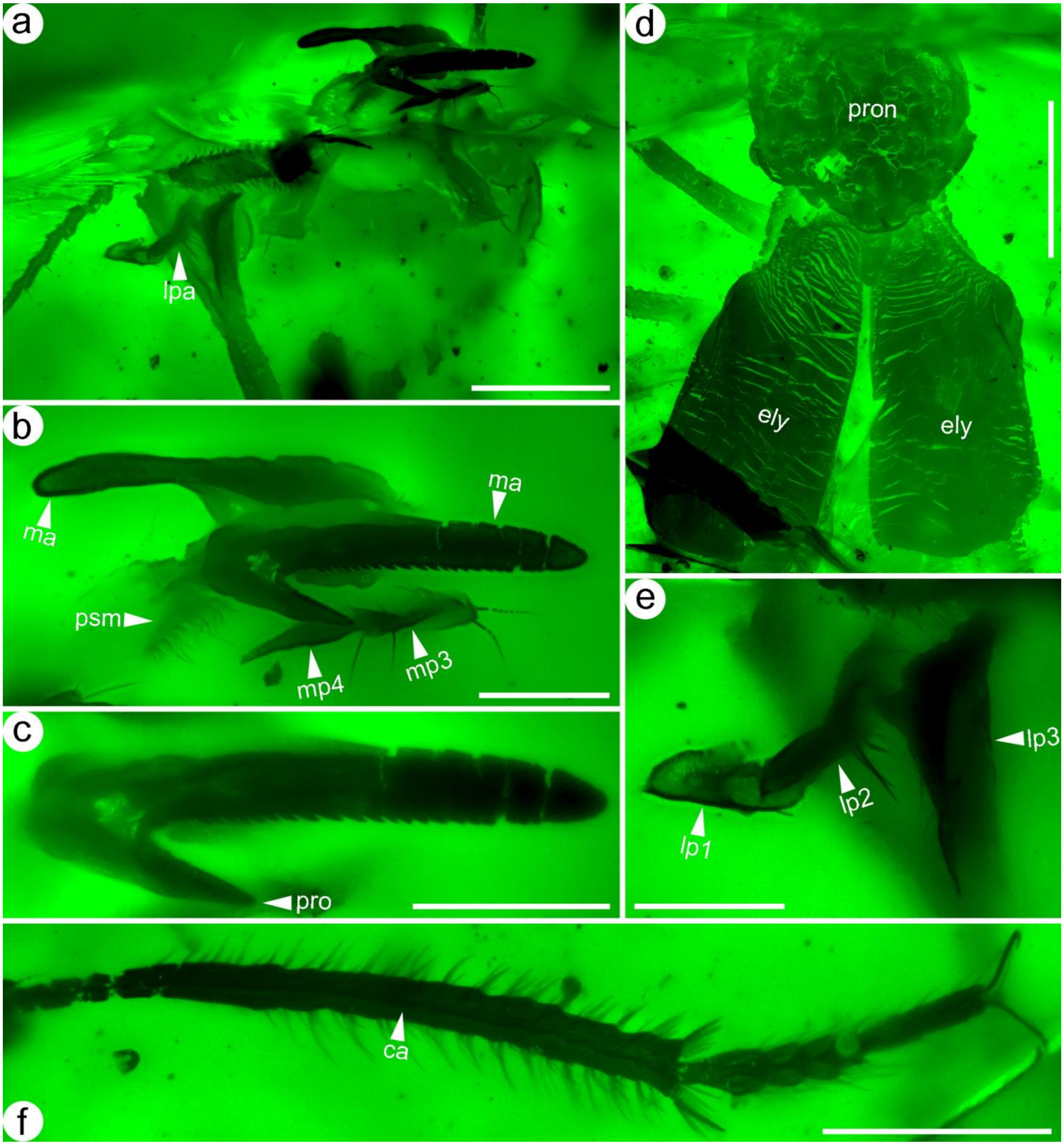

Supplementary Figure 8 | Mycophagous oxyporine rove beetles in mid-Cretaceous amber from northern Myanmar, Taxon 3, NIGP160556, under fluorescence. (**a**) Ventral view of head. (**b**) Enlargement of **a**, showing mandibles, pseudomola and left maxillary palpus. (**c**) Enlargement of **b**, showing right jagged mandible. (**d**) Enlargement of pronotum and elytra. (**e**) Enlargement of **a**, showing right labial palpus. (**f**) Right middle leg, showing keeled tibia. Abbreviations: ca, carina; ely, elytron; ma, mandible; mp, maxillary palpomere; pro, process; pron, pronotum; psm, pseudomola. Scale bars, 500 μm in **a** and **f**; 1 mm in **d**; 200 μm in others.

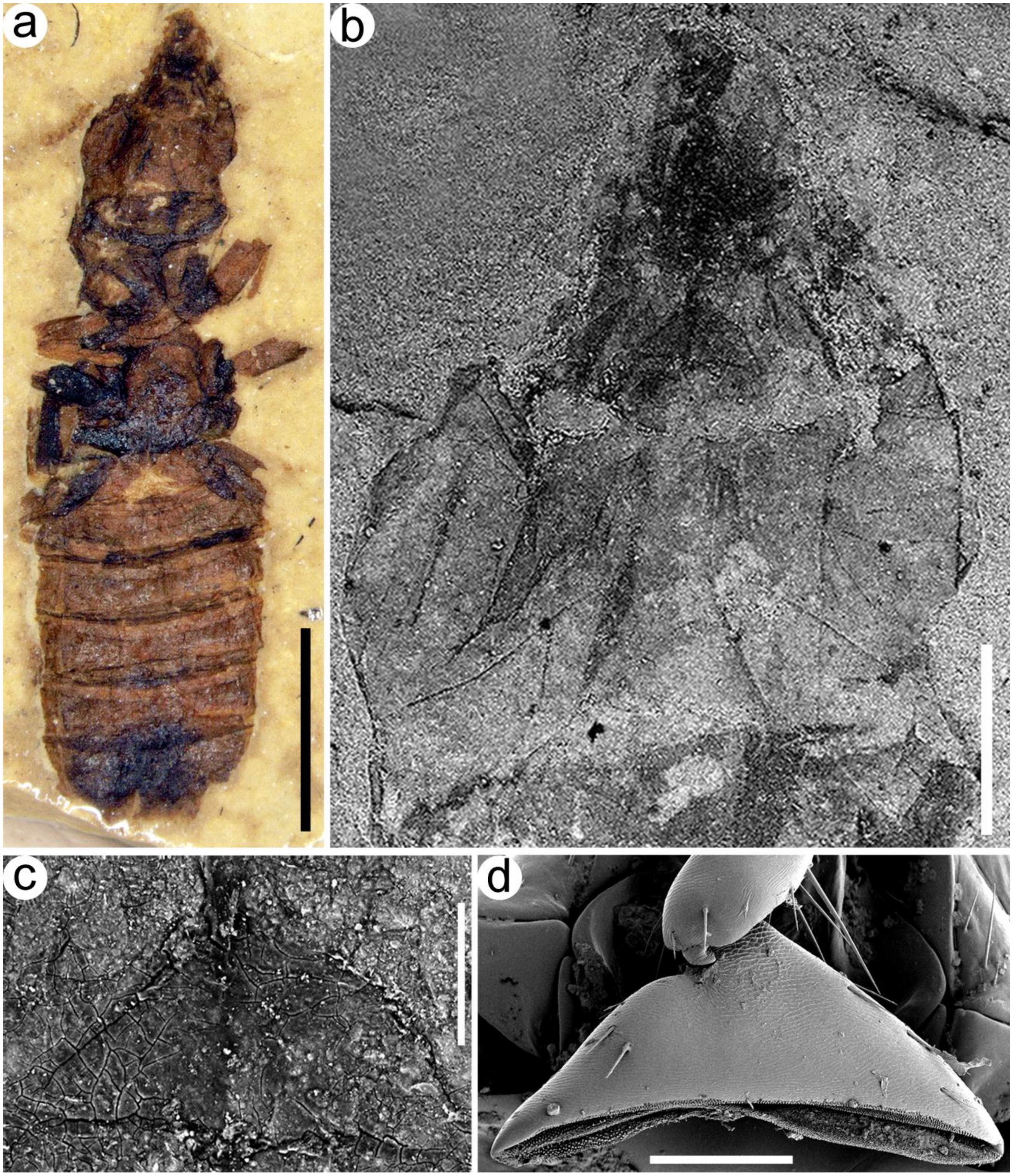

Supplementary Figure 9 | Mycophagous oxyporine rove beetles from Early Cretaceous Yixian Formation of northeastern China and comparison of apical labial palpomere between extinct and extant taxa. a under fluorescence, others under scanning electron microscope. (**a**) *Cretoxyporus extraneus* Cai and Huang, habitus of holotype, NIGP153699. (**b**) SEM of **a**, mainly showing ventral structures of head, including large mandibles. (**c**) Enlargement of **b**, showing the large and strongly securiform apical palpomere. (**d**) Enlargement of apical palpomere of extant *Oxyporus* sp. Scale bars: 2 mm in **a**; 500 μm in **b**; 100 μm in **c**. 200 μm in **d**.

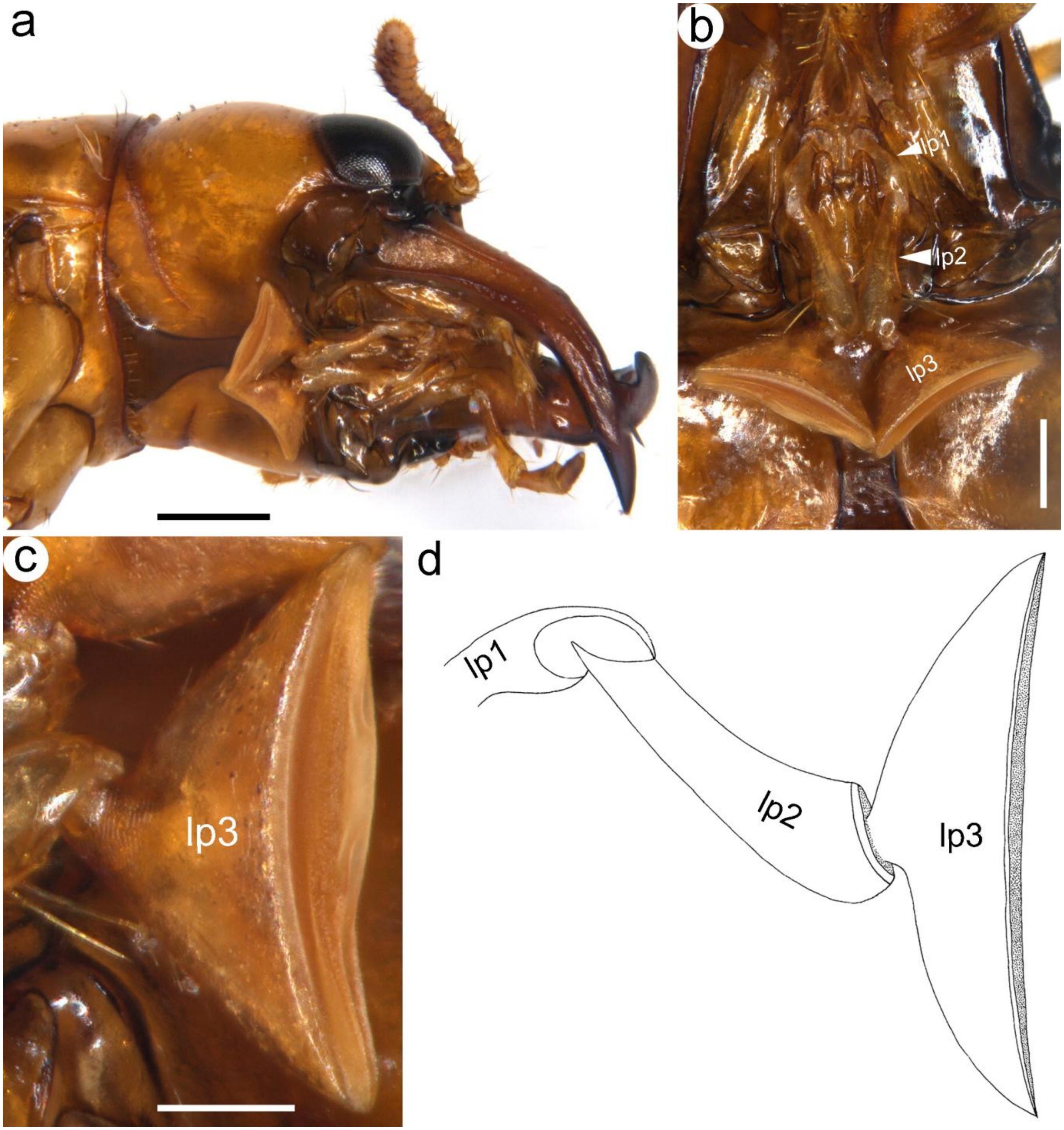

Supplementary Figure 10 | Extant mycophagous oxyporine rove beetles, *Oxyporus* sp. (**a**) Lateroventral view of head, showing details of specialized mouthparts. (**b**) Enlargement of **a**, showing details of labial palpi. (**c**) Enlargement of **b**, showing strongly securiform apical palpomere. (**d**) Line drawing of labial palpus. Abbreviation: lp, labial palpomere. Scale bars: 2 mm in **a**; 1 mm in **b**; 500 μm in **c**.

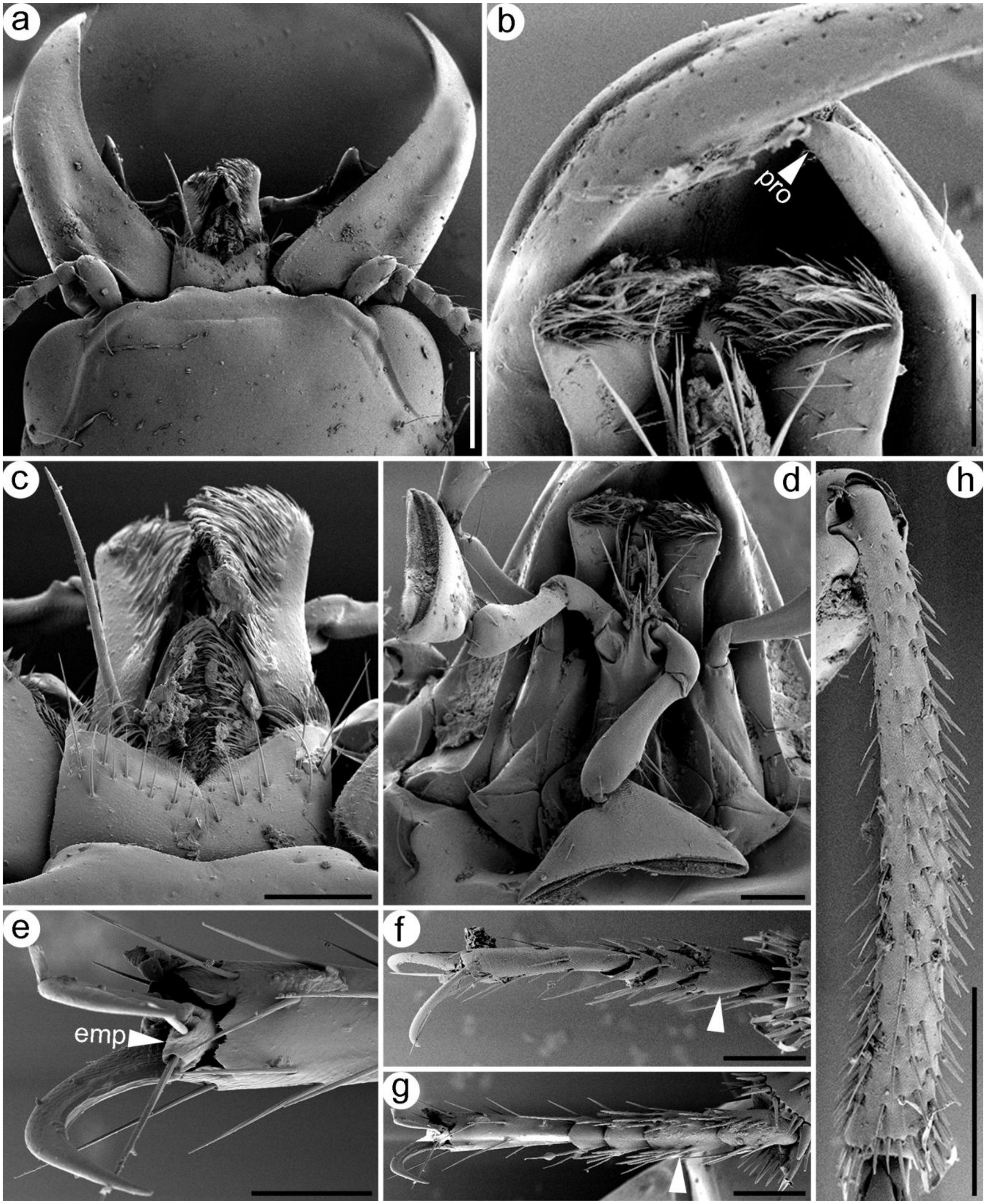

Supplementary Figure 11 | Details of extant oxyporine rove beetle, *Oxyporus* sp., SEM. (**a**) Anterior part of head, showing large mandibles. (**b**) Ventral view of crossed mandibles, indicating a small process. (**c**) Details of labium and brush-like pseudomola. (**d**) Labial palpi. (**e**) Enlargement of metatarsomere 5, showing elongate claws and empodium. (**f**) Mesotarsus, with elongate tarsomere 2 indicated. (**g**) Metatarsus, with elongate tarsomere 2 indicated. (**h**) Right mesotibia. Abbreviations: emp, empodium; pro, process. Scale bars, 500 μm in **a** and **h**; 100 μm in **e**; 200 μm in others.

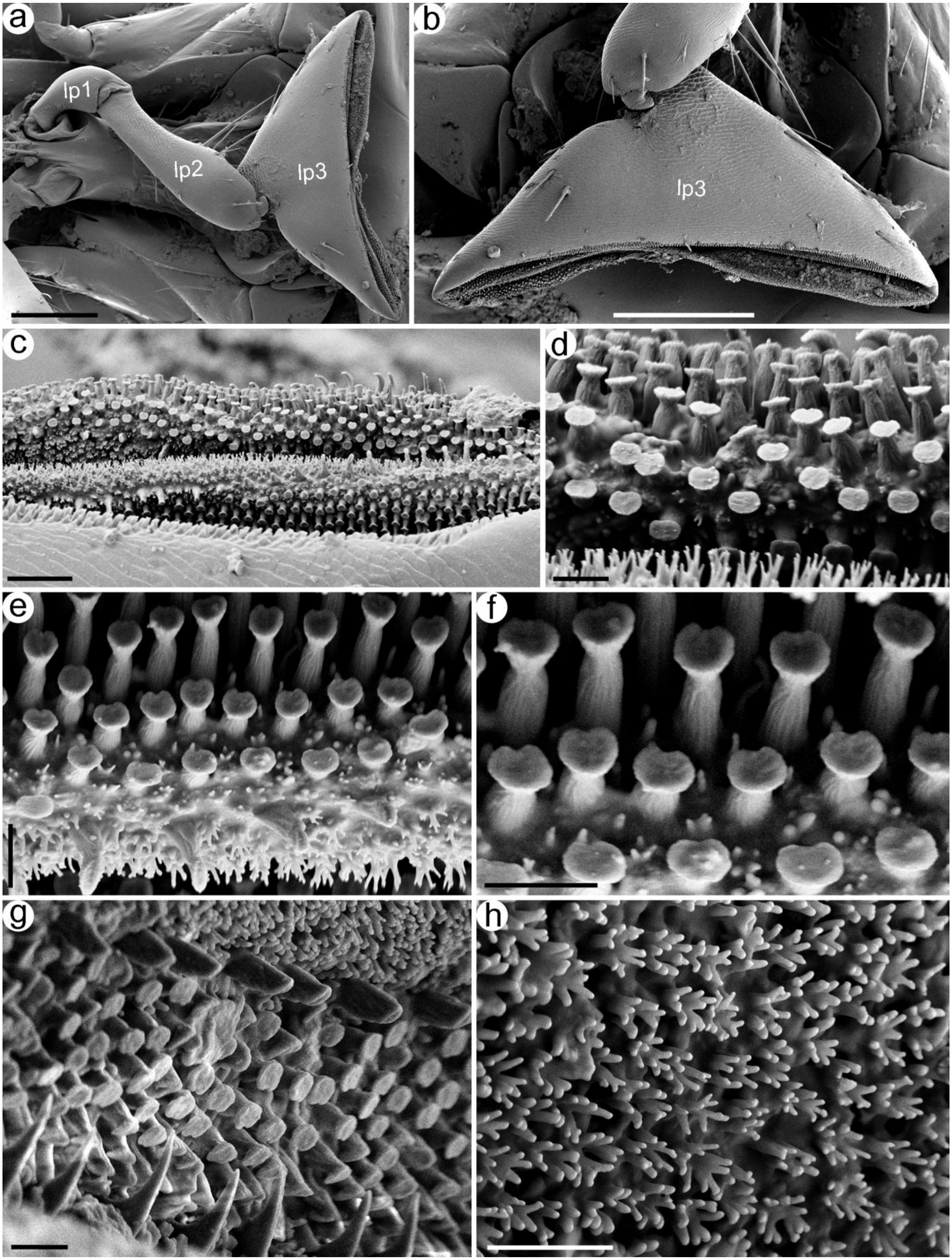
a
lp1
lp2
lp3
b
lp3
c
d
e
f
g
h

Supplementary Figure 12 | Details of mouthparts of extant mycophagous oxyporine rove beetle, *Oxyporus* sp., SEM. (**a**) Left labial palpus. (**b**) Enlargement of **a**, showing the apical labial palpomere. (**c**) Enlargement of **b**, showing fine, dense, mainly peg-like sensory organs on the apex of palpomere. (**d**) Enlargement of **c**, showing details of peg-like sensory organs. (**e**) Enlargement of **c**, showing both peg-like and villiform sensory organs. (**f**) Enlargement of **e**, showing peg-like sensory organs. (**g**) Enlargement of **c**, showing four different types of sensilla, including villiform, digitiform, peg-like and acronychius sensilla**. (h**) Details of villiform sensory organs. Abbreviations: lp, labial palpomere. Scale bars, 200 μm in **a** and **b**; 20 μm in **c**; 5 μm in others.

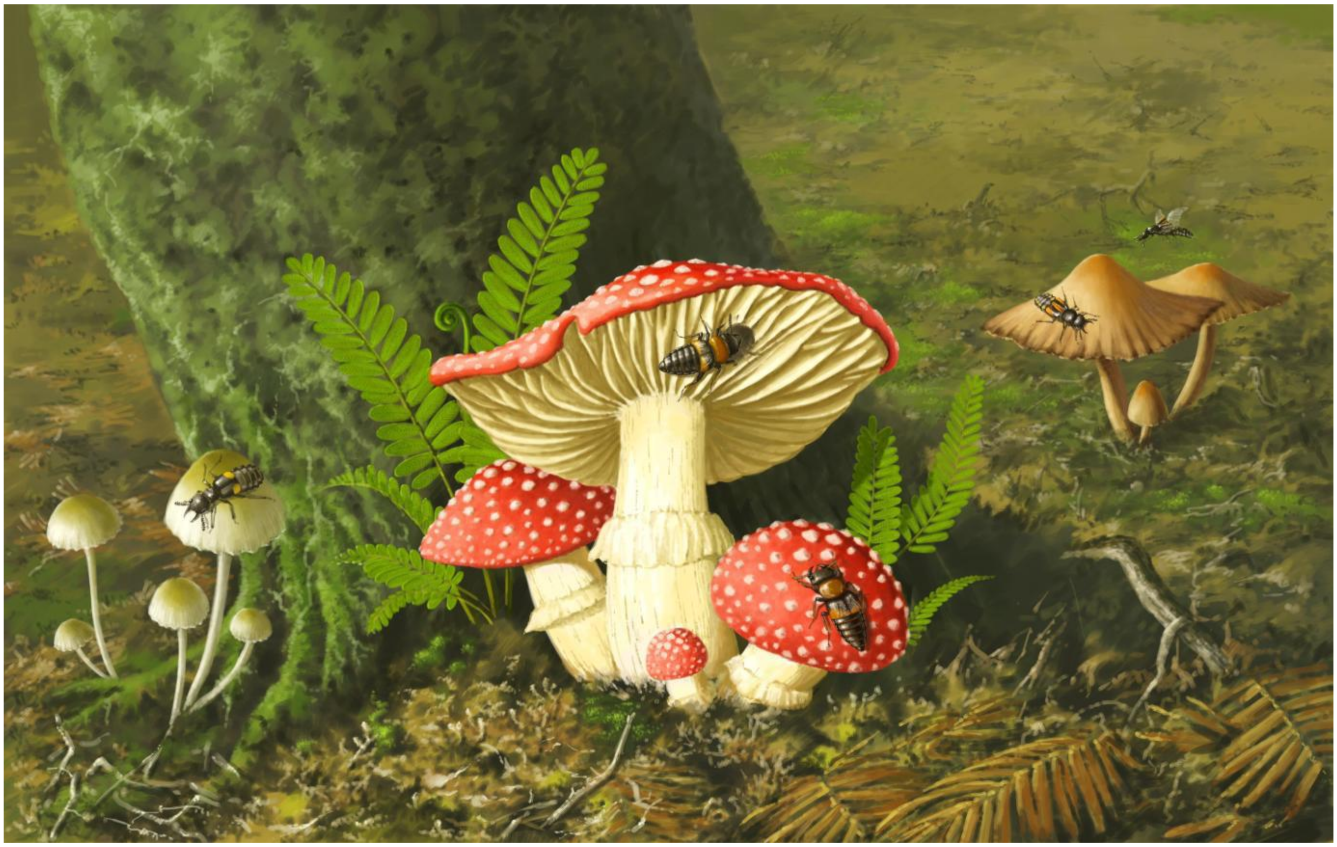

Supplementary Figure 13 | Ecological reconstruction of diverse mycophagous oxyporine rove beetles and mushrooms in the Cretaceous; image courtesy of Jie Sun.

Supplementary Table 1 | Checklist of known fossil agarics.

**Number**	**Fossil Agarics**	**Size & Shape**	**Systematic Placement**	**Deposit**	**Age**	**Source**
**1**	*Coprinites dominicana* Poinar & Singer, 1990	Pileus 3.5 mm in largest diameter; convex-shaped with a small central depression	Order Agaricales, Family Coprinaceae*	Dominican amber	~20 Ma	ref. 1
**2**	*Archaeomarasmius leggetti* Hibbett, Grimaldi & Donoghue, 1997	Pileus 3.2–6.0 mm in diameter; circular, plano-convex, radially sulcate; margin incurved	Order Agaricales, Family Tricholomataceae**	New Jersey amber	~90 Ma	ref. 2
**3**	*Protomycena electra* Hibbett, Grimaldi & Donoghue, 1997	Pileus 5 mm in diameter, 4 mm in height; circular, convex; margin slightly flared; context thin	Order Agaricales, Family Tricholomataceae***	Dominican amber	~20 Ma	ref. 2
**4**	*Aureofungus yaniguaensis* Hibbett et al., 2003	Pileus 3 mm in diameter; convex, with a broad raised center, glabrous or minutely textured; margin incurved, striated	Order Agaricales, Family incertae sedis	Dominican amber	~20 Ma	ref. 3
**5**	*Palaeoagaracites antiquus* Poinar & Buckley, 2007	Pileus 2.2 mm in diameter; convex, radially furrowed, hairy; margin slightly incurved	Order Agaricales, Family incertae sedis	Burmese amber	~99 Ma	ref. 4
**6**	Taxon A	See above	Order Agaricales, Family incertae sedis	Burmese amber	~99 Ma	Present study
**7**	Taxon B	See above	Order Agaricales, Family incertae sedis	Burmese amber	~99 Ma	Present study
**8**	Taxon C	See above	Order Agaricales, Family incertae sedis	Burmese amber	~99 Ma	Present study
**9**	Taxon D	See above	Order Agaricales, Family incertae sedis	Burmese amber	~99 Ma	Present study

**Coprinites* was originally placed in Coprinaceae, but questioned by Hibbet *et al.*[2], who suggested that *Coprinites* could represent either the Agaricaceae or a nondeliquescent member of the Coprinaceae.

***Archaeomarasmius* was suggested to be closely related to the modern genera *Marasmius* and *Marasmiellus*, which were traditionally placed in the polyphyletic family Tricholomataceae. However, both genera have been segregated into Marasmiaceae.

****Protomycena* was compared with extant *Mycena* and placed in the Myceneae, a subfamily was attributed to the family Tricholomataceae. *Mycena* is currently classified in the family Mycenaceae, suggesting that *Protomycena* probably belongs to Mycenaceae, rather than Tricholomataceae.

Supplementary Table 2 | Oldest fossil records of subfamilies of Staphylinidae.

Subfamily	Occurrence of oldest known fossil	Presence in Cretaceous?	Source
Aleocharinae	earliest Late Cretaceous (~99 Ma); Burmese amber	Yes	ref. 5
Apateticinae	No fossils known	No	–
Dasycerinae	earliest Late Cretaceous (~99 Ma); Burmese amber	Yes	ref. 6
Empelinae	No fossils known	No	–
Euaesthetinae	Early Cretaceous (~125–135 Ma); Lebanese amber	Yes	ref. 7
Glypholomatinae	Middle Jurassic Haifanggou Formation (~165 Ma); Daohugou beds	No	ref. 8
Habrocerinae	No fossils known	No	–
Leptotyphlinae	No fossils known	No	–
Megalopsidiinae	earliest Late Cretaceous (~99 Ma); Burmese amber	Yes	ref. 9
Micropeplinae	earliest Late Cretaceous (~99 Ma); Burmese amber	Yes	ref. 10
Microsilphinae	No fossils known	No	–
Neophoninae	No fossils known	No	–
Olisthaerinae	Middle Jurassic Haifanggou Formation (~165 Ma); Daohugou beds	No	ref. 11
Omaliinae	Middle Jurassic Haifanggou Formation (~165 Ma); Daohugou beds	Yes	ref. 12
Osoriinae	earliest Late Cretaceous (~99 Ma); Burmese amber	Yes	ref. 13
Oxyporinae	Early Cretceous Yixian Formation (~125 Ma); Jehol biota	Yes	ref. 14
Oxytelinae	Late Jurassic Karabastau Formation, Karatau (~150 Ma)	Yes	ref. 15
Paederinae	Early Cretceous Yixian Formation (~125 Ma); Jehol biota	Yes	ref. 16
Phloeocharinae	Late Cretceous (~90 Ma); New Jersey amber	Yes	ref. 17
Piestinae	Early Cretceous Yixian Formation (~125 Ma); Jehol biota	Yes	ref. 18
Proteininae	earliest Late Cretaceous (~99 Ma); Burmese amber	Yes	ref. 19
Protopselaphinae	earliest Late Cretaceous (~99 Ma); Burmese amber	Yes	Cai, personal observation
Pselaphinae	Early Cretaceous (~110 Ma); Spanish amber	Yes	ref. 20
Pseudopsinae	No fossils known	No	–
Scaphidiinae	earliest Late Cretaceous (~99 Ma); Burmese amber	No	Cai, personal observation
Scydmaeninae	Early Cretaceous (~110 Ma); Spanish amber	Yes	ref. 20
Solieriinae	earliest Late Cretaceous (~99 Ma); Burmese amber	Yes	ref. 21
Staphylininae	Early Cretceous Yixian Formation (~125 Ma); Jehol biota	Yes	ref. 16
Steninae	earliest Late Cretaceous (~100 Ma); French amber	Yes	ref. 22
Tachyporinae	Late Jurassic Karabastau Formation, Karatau (~150 Ma)	Yes	ref. 15
Trigonurinae	Late Jurassic Karabastau Formation, Karatau (~150 Ma)	No	ref. 15
Trichophyinae	No fossils known	No	–

Supplementary Note 1

Description of Mushrooms in Burmese Amber

Kingdom: Fungi

Phylum: Basidiomycota

Class: Agaricomycetes

Order: Agaricales

Family: Marasmiaceae

Taxon A

(Fig. 1a, b, and Supplementary Figs 1a and 2a)

Material. NIGP164520; nearly completely-preserved, with an intact cap possessing characterized gills; stalk broken, with basal portion preserved; deposited in the Nanjing Institute of Geology and Palaeontology, Chinese Academy of Sciences.

Locality and age. Preserved in Burmese amber (Burmite) from Hukawng Valley, northern Myanmar; earliest Cenomaniaas aboven, absolute age 98.79 ± 0.62 million years (myr) ago established by U–Pb dating of zircons from the rind of the unprocessed amber.

Description. Basidiomata dark brown. Pileus 2.86 mm in largest diameter, plano-convex, circular, thin-fleshed, radially sulcate, depressed in middle; margin strongly incurved (Supplementary Fig. 2b). Lamellae sub-distant, with ca. 13 primary lamellae extending from stipe apex to pileus margin and ca. 13 lamellulae (of one length). Stipe slender, base broken, largest diameter 0.22 mm, insertion central, cylindrical.

Taxon B

(Fig. 1c and Supplementary Figs 1c and 2c)

Material. NIGP164521 and NIGP164522. NIGP164521 with complete cap and partial stalk; NIGP164522 with broken cap and stalk; deposited in the Nanjing Institute of Geology and Palaeontology, Chinese Academy of Sciences.

Locality and age. Preserved in Burmese amber (Burmite) from Hukawng Valley, northern Myanmar; earliest Cenomanian (98.79 ± 0.62 myr).

Description. Basidiomata dark brown. Pileus 2.94 mm in diameter, strongly plano-convex (Supplementary Fig. 2e), circular, thin-fleshed, slightly radially sulcate (Supplementary Fig. 2d); margin slightly incurved; surface minutely textured. Lamellae sub-distant, with about 20 visible lamellae, attached to stipe apex. Stipe slender, slightly curved, base broken, diameter 0.35 mm, insertion central, cylindrical.

Family: incertae sedis

Taxon C

(Fig. 1d and Supplementary Figs 1b and 3c)

Material. NIGP164523; mainly preserved as cap; stalk with only basal part visible; deposited in the Nanjing Institute of Geology and Palaeontology, Chinese Academy of Sciences.

Locality and age. Preserved in Burmese amber (Burmite) from Hukawng Valley, northern Myanmar; earliest Cenomanian (98.79 ±0.62 myr).

Description. Basidiomata light brown. Pileus 3.92 mm in largest diameter, slightly convex, sub-oval, thin-fleshed, not sulcate, shallowly depressed in middle; margin not incurved; surface coarsely textured. Lamellae close (Supplementary Fig. 3e), with about 38 visible lamellae and only 10 extend from margin to stipe. Stipe with only apex preserved, insertion sub-marginal, cylindrical (Supplementary Fig. 3d).

Taxon D

(Fig. 1e and Supplementary Figs 1d and 3a, b)

Material. NIGP164524; nearly completely preserved, with a piece of elongate plant remains covering the cap from ventral view; deposited in the Nanjing Institute of Geology and Palaeontology, Chinese Academy of Sciences.

Locality and age. Preserved in Burmese amber (Burmite) from Hukawng Valley, northern Myanmar; earliest Cenomanian (98.79 ±0.62 myr).

Description. Basidiomata dark. Pileus 2.65 mm in largest diameter, plano-convex, thin-fleshed, radially sulcate; margin plicate-pectinate, not incurved; surface minutely textured. Lamellae sub-distant, with about 16 visible lamellae. Stipe very short and stout, base preserved, diameter 0.31 mm, insertion sub-marginal, cylindrical.

Supplementary Note 2

Description of Mycophagous Oxyporine Rove Beetles in Burmese Amber

Kingdom: Animalia

Phylum: Arthropoda

Class: Insecta

Order: Coleoptera

Family: Staphylinidae

Subfamily: Oxyporinae

Genus: *Oxyporus* Fabricius, 1775

Taxon 1

(Fig. 2a and Supplementary Figs 4 and 5)

Material. NIGP164525, NIGP164526, NIGP164527; three conspecific individuals preserved in one amber piece prior to preparation (cutting); NIGP164525 and NIGP164526 preserved in one piece after preparation (Supplementary Fig. 4a); all specimens completely preserved, except for the right mesotarsus of NIGP164527; deposited in the Nanjing Institute of Geology and Palaeontology, Chinese Academy of Sciences.

Locality and age. Preserved in Burmese amber (Burmite) from Hukawng Valley, northern Myanmar; earliest Cenomanian (98.79 ± 0.62 myr).

Diagnostic description. Body moderate, 6.4–7.7 mm long, mostly yellow (except elytral apex); antenna with antennomeres 6–10 strongly transverse, broadly glabrous along axis (of the subgenus *Oxyporus* type; Supplementary Fig. 5b); infraorbital ridge absent; pronotum with a posteromedian tubercle (Supplementary Fig. 5c); elytron impunctate, bicolored, with a small, black and transverse mark on outer apical angle (Supplementary Fig. 5d); mesocoxae widely separated by large metaventral anterior process. Hind wing well developed (Supplementary Fig. 5g).

Remarks: Taxon 1 is undoubtedly placed in the extant Oxyporinae based on the following combination of characters: antenna inserted at sides of head near the eyes; large and strongly protruding mandible (Supplementary Fig. 5a, b); large conical procoxa; transverse metacoxa; tarsal formula 5-5-5 (Supplementary Fig. 5h, i); abdomen with six visible sterna; and two pairs of paratergites each on abdominal segments III–VII. Taxon 1 can be placed in the extant genus *Oxyporus* based on its general habitus, bicolored elytron, absence of infraorbital ridge, and mesocoxae widely separated by large

metaventral anterior process. It can be attributed to the extant subgenus *Oxyporus* by the characteristic antennal morphology (antennomeres 6–10 strongly transverse, broadly glabrous along axis). Taxon 1 is morphologically very similar to some extant *Oxyporus* representatives, but is differs in having a posteromedian tubercule on pronotum, impunctate elytra, and the colour, shape and size of elytral markings.

Taxon 2

(Fig. 2c and Supplementary Fig. 6)

Material. NIGP164528, a nearly complete adult with the head slightly downcurved; median portion of pronotum broken; deposited in the Nanjing Institute of Geology and Palaeontology, Chinese Academy of Sciences.

Locality and age. Preserved in Burmese amber (Burmite) from Hukawng Valley, northern Myanmar; earliest Cenomanian (98.79 ± 0.62 myr).

Diagnostic description. Body very small, 4.5 mm long, mostly brown and black, head and pronotum black, elytra and abdomen bicolored (Supplementary Fig. 6b, c); mandible elongate (Supplementary Fig. 6d); antenna with antennomeres 6–10 strongly transverse, broadly glabrous along axis (of the subgenus *Oxyporus* type); infraorbital ridge absent; pronotum without median tubercle; elytron impunctate, bicolored, with a large, black and longitudinal mark on outer apical angle, and an elongate distally-tapered mark along sutural margin (Supplementary Fig. 6c); mesocoxae widely separated by large metaventral anterior process. Hind wing well developed (Supplementary Fig. 6c).

Remarks: Similar to Taxon 1, this taxon is undoubtedly a member of the extant genus *Oxyporus* based on its large and strongly protruding mandible, large conical procoxa; tarsal formula 5-5-5 (Supplementary Fig. 6e, f); and two pairs of paratergites on abdominal segments III–VII. Moreover, it can be also attributed to the subgenus *Oxyporus* by the characteristic antennal morphology (antennomeres 6–10 strongly transverse, broadly glabrous along axis). Taxon 2 is similar to some extant *Oxyporus* species, but is differs in having a much smaller body size, bicolored elytron and abdomen, impunctate elytra, and the colour, shape and size of elytral markings.

Taxon 3

(Fig. 2c and Supplementary Figs 7 and 8)

Material. NIGP160556, a complete adult with the body slightly distorted and broken; some structures,

such as the labial palpi, detached from the mouthparts, with one labial palpus below the ventral side of right eye, and the other before the left mandible; deposited in the Nanjing Institute of Geology and Palaeontology, Chinese Academy of Sciences.

Locality and age. Preserved in Burmese amber (Burmite) from Hukawng Valley, northern Myanmar; earliest Cenomanian (98.79 ± 0.62 myr).

Diagnostic description. Body moderate, 7.3 mm long, black (including elytra); right mandible jagged-edged with a sharp process near base (Supplementary Fig. 8b, c); antenna with antennomeres 6–10 strongly transverse, broadly glabrous along axis (of the subgenus *Oxyporus* type); infraorbital ridge absent; pronotum without median tubercle; elytron impunctate, unicolored (Supplementary Fig. 8d); mesocoxae widely separated by large metaventral anterior process; tibia keeled (Supplementary Figs 7b, c and 8f); mesotarsus with first tarsomere as long as the second (Supplementary Fig. 7d). Empodium with 2 long setae (Supplementary Fig. 7e).

Remarks: Taxon 3 can be unambiguously placed in Oxyporinae based on its large and strongly protruding mandible, large conical procoxa; transverse metacoxa; 5-segmented mesotarsus, and 6-segmented abdomen with two pairs of paratergites. The taxon resembles *Oxyporus* species in general habitus, but significantly differs by the presence of impunctate elytron, distinctly keeled tibia, and tarsomere 1 as long as the second. In particular, the keeled tibiae are apparently confined to Apateticinae and the related *Mesapatetica* Cai et al., Piestinae, some basal Oxytelinae (*Platydeleaster* Schülke and *Protodeleaster* Cai et al.), and some Scaphidiinae. In *Oxyporus*, the basal tarsomeres (tarsomere 1) are distinctly smaller and shorter than tarsomere 2. However, the basal tarsomere is as long as the second in Taxon 3, making it very distinctive among oxyporines. Therefore, we suggest that taxon 3 represents a new genus belonging to the extant Oxyporinae.

Supplementary Note 3

Evolution of mycophagy in Staphylinidae

Most extant beetle families are thought to have originated in the Cretaceous[23,24] and that diet diversification in Coleoptera was well underway, taking advantage of fully functional ecosystems[25,26]. Of about 60 modern families of beetles known from the Cretaceous[27], those with extant members associated with large-bodied fungi include Ptiliidae, Eucinetidae, Trogossitidae, Nitidulidae, Tetratomidae, Melandryidae, Mordellidae, Tenebrionidae, and Anthribidae. Taxonomic diversity[16] is mirrored in Mesozoic staphylinids, the largest family of the Order Coleoptera[28], which was also diverse in the Cretaceous and consistied of 20 of the 32 extant subfamilies (Supplementary Table 2) belonging to predominantly predatory, mycophagous, and saprophagous lineages[29]. Definitive mushroom feeding by adults and larvae occurs in Oxyporinae and Scaphidiinae (an unconfirmed Upper Jurassic fossil has been identified from the Solenhofen deposits by Weyenbergh[30]; a definitive scaphidiine has recently been discovered from Burmese amber). Saprophagy may be the primitive mechanism for Staphyliniformia as a whole[31,32], which is corroborated by presumed behaviors of Jurassic staphylinids, that also included fungus-feeding Glypholomatinae[8]. Phylogenetic data on staphyliniforms corroborate fossil evidence indicating that staphylinid diversification began in the Jurassic[33]. Character analyses reveal a high number of phylogenetic shifts among various diets and microhabitats, more commonly from leaf litter habitats to subcortical (saproxylic) or to periaquatic microhabitats with fewer shifts from leaf litter to carrion and to fungi[34].

The exact phylogenetic placement of Oxyporinae remains controversial[28,34], complicating the exact nature of the origin of mushroom feeding in the group. Oxyporinae are placed in the predatory Staphylinine Group of subfamilies[35], usually in a basal position relative to Megalopsidiinae, Pseudopsinae, Steninae, Euaesthetinae[36,37]. This staphylinid subgroup is dated to the Cretaceous[38] and contains very diverse prey capture strategies, including the use of a sticky-harpoon method by Steninae that have a protrusible labium[39]. Well-sampled phylogenetic studies place Oxyporinae with low statistical support outside the Staphylinine Group, for example, as sister taxon to Osoriinae[28] or sister to soil-dwelling Leptophylinae[34]. Though the long-held view that Oxyporinae arose from a predatory ancestor remains ambiguous based on recent phylogenetic studies, by the Cretaceous, staphylinid feeding mechanisms had well-diversified.

Supplementary References

1. Poinar, G. O. & Singer, R. Upper Eocene gilled mushroom from the Dominican Republic. *Science* **248**, 1099–1101 (1990).
2. Hibbett, D., Grimaldi, D. & Donoghue, M. Fossil mushrooms from Miocene and Cretaceous ambers and the evolution of Homobasidiomycetes. *Am. J. Bot.* **84**, 981–981 (1997).
3. Hibbett, D. S., Binder, M., Wang, Z. & Goldman, Y. Another fossil agaric from Dominican amber. *Mycologia* **95**, 685–687 (2003).
4. Poinar, G. O. & Buckley, R. Evidence of mycoparasitism and hypermycoparasitism in Early Cretaceous amber. *Mycol. Res.* **111**, 503–506 (2007).
5. Cai, C. & Huang D. The oldest aleocharine rove beetle (Coleoptera, Staphylinidae) in Cretaceous Burmese amber and its implications for the early evolution of the basal group of hyper-diverse Aleocharinae. *Gondwana Res.* **28**, 1579–1584 (2015).
6. Yamamoto, S. The first fossil of dasycerine rove beetle (Coleoptera: Staphylinidae) from Upper Cretaceous Burmese amber: Phylogenetic implications for the omaliine group subfamilies. *Cretaceous Res.* **58**, 63–68 (2016).
7. Lefebvre, F., Vincent, B., Azar, D. & Nel, A. The oldest beetle of the Euaesthetinae (Staphylinidae) from Early Cretaceous Lebanese amber. *Cretaceous Res.* **26**, 207–211 (2005).
8. Cai, C., Huang, D., Thayer, M. K. & Newton, A. F. Jr. Glypholomatine rove beetles (Coleoptera: Staphylinidae): a southern hemisphere Recent group recorded from the Middle Jurassic of China. *J. Kansas Entomol. Soc.* **85**, 239–244 (2012).
9. Yamamoto, S. & Solodovnikov, A. The first fossil Megalopsidiinae (Coleoptera: Staphylinidae) from Upper Cretaceous Burmese amber and its potential for understanding basal relationships of rove beetles. *Cretaceous Res* **59**, 140–146. (2016).
10. Cai, C. & Huang D. The oldest micropepline beetle from Cretaceous Burmese amber and its phylogenetic implications (Coleoptera: Staphylinidae). *Naturwissenschaften* **101**, 813–817 (2014).
11. Cai, C., Beattie, R. & Huang D. Jurassic olisthaerine rove beetles (Coleoptera: Staphylinidae): 165 million years of morphological and probably behavioral stasis. *Gondwana Res.* **28**, 425–431 (2015).
12. Cai, C. & Huang D. *Sinanthobium daohugouense*, a tiny new omaliine rove beetle from the Middle Jurassic of China (Coleoptera, Staphylinidae). *Canadian Entomol.* **145**, 496–500 (2013).
13. Cai, C. & Huang D. The oldest osoriine rove beetle from Cretaceous Burmese amber (Coleoptera: Staphylinidae). *Cretaceous Res.* **52**, 495–500 (2015).
14. Cai, C. & Huang, D. Diverse oxyporine rove beetles from the Early Cretaceous of China (Coleoptera: Staphylinidae). *Syst. Entomol.* **39**, 500–505 (2014).

15. Tikhomirova, A. L. Staphylinid beetles of the Jurassic of the Karatau (Coleoptera, Staphylinidae). in *Jurassic Insects of the Karatau* (ed. Rohdendorf, B. B.) 139–154 (Akademiya Nauk SSSR, 1968) (in Russian).
16. Solodovnikov, A., Yue, Y., Tarasov, S. & Ren, D. Extinct and extant rove beetles meet in the matrix: early Cretaceous fossils shed light on the evolution of a hyperdiverse insect lineage (Coleoptera: Staphylinidae: Staphylininae). *Cladistics* **29**, 360–403 (2013).
17. Chatzimanolis, S., Newton, A. F., Soriano, C. & Engel, M. S. Remarkable stasis in a phloeocharine rove beetle from the Late Cretaceous of New Jersey (Coleoptera, Staphylinidae). *J. Paleontol.* **87**, 177–182 (2013).
18. Yue, Y., Gu, J., Yang, Q., Wang, J. & Ren, D. The first fossil species of subfamily Piestinae (Coleoptera: Staphylinidae) from the Lower Cretaceous of China. *Cretaceous Res*. **63**, 63–67 (2016).
19. Cai, C. *et al.* Specialized proteinine rove beetles shed light on insect–fungal associations in the Cretaceous. *Proc. R. Soc. B* **283**, 20161439 (2016).
20. Peris, D., Chatzimanolis, S. & Delclòs, X. Diversity of rove beetles (Coleoptera: Staphylinidae) in early Cretaceous Spanish amber. *Cretaceous Res*. **48**, 85–95 (2014).
21. Thayer, M. K., Newton, A. F. & Chatzimanolis, S. *Prosolierius*, a new mid-Cretaceous genus of Solieriinae (Coleoptera: Staphylinidae) with three new species from Burmese amber. *Cretaceous Res.* **34**, 124–134 (2012).
22. Schlüter, T. Zur Systematik und Palökologie harzkonservierter Arthropoda einer Taphozönose aus dem Cenomanium von NW-Frankreich. A: Geologie und Paläontologie. *Berliner Geowissenschaftliche Abhandlungen* **9**, 1–150 (1978).
23. Grimaldi, D. A. & Engel, M. S. *Evolution of the insects*. (Cambridge University Press, 2005).
24. Hunt, T. *et al*. A comprehensive phylogeny of beetles reveals the evolutionary origins of a superradiation. *Science* **318**, 1913–1916 (2007).
25. Schmidt, A. R., Dörfelt, H., Struwe, S., & Perrichot, V. Evidence for fungivory in Cretaceous amber forests from Gondwana and Laurasia. *Palaeontographica Abteilung B* **283**, 157–173 (2010).
26. Adl, S. *et al*. Reconstructing the soil food web of a 100 million-year-old forest: The case of the mid-Cretaceous fossils in the amber of Charentes (SW France). *Soil Biol. Biochem.*, **43**, 726–735 (2011).
27. EDNA. Fossil Insects Database, hosted by the Palaeontological Association. Available online at http://edna.palass-hosting.org (accessed 10 July 2016) (2016).
28. Grebennikov, V. V. & Newton, A. F. Good-bye Scydmaenidae, or why the ant-like stone beetles

should become megadiverse Staphylinidae sensu latissimo (Coleoptera). *Eur. J. Entomol.* **106**, 275−301 (2009).

29. Thayer, M. K. Staphylinidae Latreille, 1802. in *Coleoptera, Beetles. Vol. 1, Morphology and Systematics (Archostemata, Adephaga, Myxophaga, Polyphaga Partim).* (eds Beutel, R. G. & Leschen, R. A. B.) in *Handbuch der Zoologie/Handbook of Zoology. Band/Vol. IV: Arthropoda: Insecta; Part 38* (eds Kristensen, N. P. & Beutel, R. G.) 296–344 (Walter de Gruyter, 2005).
30. Weyenbergh, H. Jr. Sur les insectes fossiles du calcaire Iithographique de la Baviere, qui se trouvent au Musee Teyler. *Archives du Musee Teyler* **2**, 247–294, 34–37 pls (1869).
31. Hansen, M. Phylogeny and classification of the staphyliniform beetle families (Coleoptera). *Biol Skr, Det K Dan Vidensk Selsk* **48**, 1–339 (1997).
32. Betz, O., Newton, A. F. & Thayer, M. K. Comparative morphology and evolutionary pathways of the mouthparts of spore-feeding Staphylinoidea (Coleoptera). *Acta Zoologica* **84**, 179–238 (2003).
33. Zhang, X. & Zhou, H.-Z. How old are the rove beetles (Insecta: Coleoptera: Staphylinidae) and their lineages? Seeking an answer with DNA. *Zool. Sci.* **30**, 490–501 (2013).
34. Mckenna, D. D. *et al*. Phylogeny and evolution of the Staphyliniformia and Scarabaeiformia: Forest litter as a stepping-stone for diversification of non-phytophagous beetles. *Syst. Entomol.* **40**, 35−60 (2015).
35. Lawrence, J. F. & Newton, A. F. Jr. Families and subfamilies of Coleoptera (with selected genera, notes, references and data on family-group names). in *Biology, Phylogeny and Classification of Coleoptera. Papers celebrating 80th Birthday of Roy A. Crowson* (eds Pakaluk, J. & Slipinski, S. A.) 779–1006 (Muzeum i Instytut Zoologii PAN, 1995).
36. Leschen, R. A. B., Newton, A. F. Larval description, adult feeding, behavior, and phylogenetic placement of *Megalopinus* (Coleoptera: Staphylinidae). *Coleopts. Bull.* **57**, 469–493 (2003).
37. Clarke, D. J. & Grebennikov, V. V. Monophyly of Euaesthetinae (Coleoptera: Staphylinidae): phylogenetic evidence from adults and larvae, review of austral genera, and new larval descriptions. *Syst. Entomol.* **34**, 346–397 (2009).
38. Clarke, D. J. & Chatzimanolis, S. Antiquity and long-term morphological stasis in a group of rove beetles (Coleoptera: Staphylinidae): Description of the oldest *Octavius* species from Cretaceous Burmese amber and a review of the "Euaesthetine subgroup" fossil record. *Cretaceous Res.* **30**, 1426–1434 (2009).
39. Koerner, L., Laumann, M., Betz, O., & Heethoff, M. Loss of the sticky harpoon–COI sequences indicate paraphyly of *Stenus* with respect to *Dianous* (Staphylinidae, Steninae). *Zoologischer Anzeiger* **252**, 337–347(2013).
